# Peer Review of “Waiting Time and Patient Satisfaction in a Subspecialty Eye Hospital Using a Mobile Data Collection Kit: Pre-Post Quality Improvement Intervention”

**DOI:** 10.2196/40444

**Published:** 2022-08-09

**Authors:** Alexandros Argyriadis

**Affiliations:** 1 School of Health Sciences Frederick University Nicosia Cyprus

**Keywords:** Waiting time, waiting list, patient satisfaction, quality improvement, clinical audit, ophthalmology, patient-centered care

*This is a peer-review report submitted for the paper “Waiting Time and Patient Satisfaction in a Subspecialty Eye Hospital Using a Mobile Data Collection Kit: Pre-Post Quality Improvement Intervention.”*

## Round 1 Review

### General Comments

This article [1] studies the waiting time and patient satisfaction in a subspecialty eye hospital in Cameroon. It is a matter of fact that hospital-waiting time is a major concern in many other countries, but it is important that this paper concentrates on Cameroon. Moreover, the article mentions the use of a mobile data collection kit conducting pre-post quality improvement intervention.

The article can be characterized as quite innovative and offers a significant connection of theory with practice.

It would be quite interesting if the authors mention the reasons why they chose Cameroon and refer to some similar recent research. Although, it is clear why this project is necessary to be studied. The objectives of the study are clear and combine waiting time and satisfaction, 2 important factors for the increase of quality-of-life indicators. The methods that were used are suitable and adequate for this project and the authors follow a correct pathway for the implementation of their work.

The research leads to the result that the use of plan-do-study-act (PDSA) led to a borderline significant reduction of 65.4 minutes in waiting time over 6 weeks and an insignificant improvement in satisfaction, suggesting that quality improvement efforts have to be maintained over a considerable period to be able to produce significant changes. The study provides a good basis for quality improvement in limited-resource settings making use of block appointment systems, with comprehensive subspecialty eye care services. We recommend shortening the patient pathway and other measures including a phasic appointment system, automated patient time monitor, robust ticketing, patient pathway supervision, standard triaging, task shifting, doctor consultation planning, patient education, and additional registration staff.

### Specific Comments

#### Minor Comments

1. It would be quite interesting if the authors mention the reasons why they chose Cameroon and refer to some similar recent research.
2. The description of the problem can be enriched with some more information.
3. A justification of why this research method was chosen can be an extra asset for this interesting work.
4. I also believe that the authors have used too many references than normal in a paper. They might decrease the number of references and stay in the most appropriate range. Too many citations are used in this paper. Most journals recommend no more than 40 references.

## References

[1] Mbwogge M, Astbury  N, Nkumbe  HE, Bunce C, Bascaran C (2022). Waiting time and patient satisfaction in a subspecialty eye hospital using a mobile data collection kit: pre-post quality improvement intervention. JMIRx Med.

